# Proteomic profiling identifies *SMARCA1* as a stage-specific epigenetic regulator of colorectal cancer metastasis through MMP modulation

**DOI:** 10.1016/j.gendis.2025.102006

**Published:** 2025-12-24

**Authors:** Chunqing Fu, Shoufeng Duan, Chengwen Zhang, Xinyu Cui, Fengxian Wang, Lichen Wang, Jinglei Hu, Tengjiao Li, Lin Li

**Affiliations:** a Shanghai Frontiers Science Center of Drug Target Identification and Delivery, School of Pharmaceutical Sciences, Shanghai Jiao Tong University, Shanghai 200240, China; b State Key Laboratory of Innovative Immunotherapy, Shanghai Jiao Tong University, Shanghai 200240, China

Colorectal cancer (CRC) ranks as the third most prevalent cancer worldwide. While epigenetic dysregulation is recognized as a key contributor to CRC progression, its specific role in metastasis remains inadequately understood. The paired SW480/SW620 cell lines (primary *vs*. lymph node metastasis from one patient) offer a powerful model for elucidating molecular alterations associated with metastatic transformation. Previous proteomic studies on these cells focused on secretory proteins or GTPases.^1^^,^^2^ However, the epigenetic regulatory networks, particularly chromatin remodelers, have yet to be thoroughly investigated. This study addresses this gap by systematically examining the differences in epigenetic regulatory proteins between SW480 and SW620 cells, aiming to identify key epigenetic factors that may underpin the metastatic process in CRC.

To investigate critical factors involved in CRC development, we established a proteomic workflow to quantitatively profile changes in epigenetic regulatory proteins in SW480 and SW620 cells (Fig. 1A). To strategically enrich epigenetic regulatory proteins, we performed subcellular fractionation to isolate nuclear chromatin (Fig. S1A), thereby increasing the specificity of subsequent proteomic analyses. After that, we employed data-dependent acquisition proteomics to initially detect spectral signatures matching 114 proteins. To expand proteome coverage, we subsequently developed a coding sequence (CDS) library for the remaining 63 undetected proteins. Using the curated spectral library, data-independent acquisition-mass spectrometry analysis enabled the quantification of 177 proteins, with each protein represented by at least one or two unique peptide signatures (Table S1). In contrast, liquid chromatography-tandem mass spectrometry analysis in data-dependent acquisition mode identified only 114 of the 177 proteins in the chromatin-enriched fraction. This highlights the superior sensitivity of our data-independent acquisition-mass spectrometry-based approach compared with traditional shotgun proteomics.

**Figure 1:**
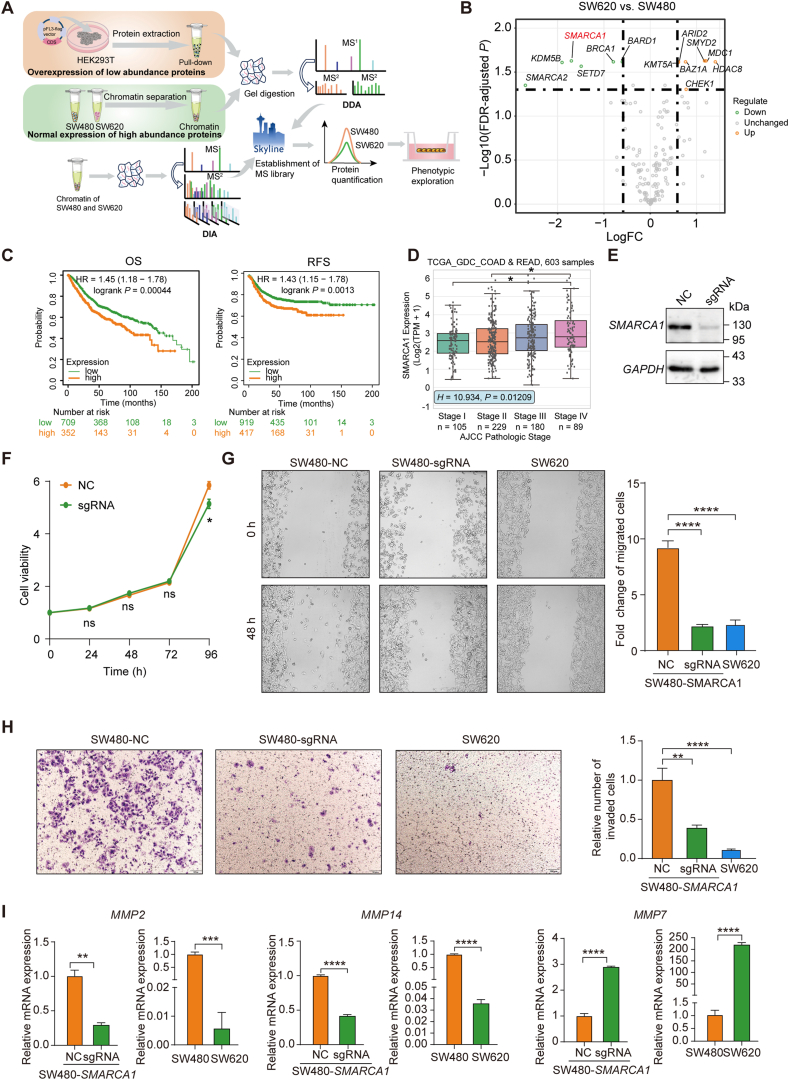
Identification of *SMARCA1* as a metastasis-associated chromatin remodeler in colorectal cancer progression. **(A)** Scheme of the experimental design. **(B)** Volcano plot analysis between SW620 and SW480 cells (|fold-change| > 1.5, FDR-adjusted *p* < 0.05), based on integrated data-dependent (DDA) and data-independent (DIA) mass spectrometry. **(C)** Kaplan–Meier analysis of overall survival (OS) and recurrence-free survival (RFS) in the TCGA colorectal cancer cohort. **(D)***SMARCA1* mRNA expression across different stages of colorectal cancer in the TCGA cohort (data combined from TCGA–COAD and TCGA–READ). Statistical significance was assessed by the Kruskal–Wallis test with Dunn's post hoc pairwise comparisons. Pairwise *p*-values were adjusted using the FDR method. ∗FDR-adjusted *p* < 0.05. **(E)***SMARCA1* knockdown in SW480 cells was achieved using the CRISPR/Cas9-sgRNA system and verified by Western blotting. **(F)** Cell proliferation of SW480 cells between wild-type and *SMARCA1* knockdown over a 0–96 h timeframe, analyzed by CCK-8 assay. **(G)** Cell migration of SW620 cells, wild-type SW480 cells, and *SMARCA1*-knockdown SW480 cells at 0–48 h was assessed using a wound-healing assay. **(H)** Cell invasion of SW620 and wild-type SW480 cells, as well as *SMARCA1*-knockdown SW480 cells, was assessed by Transwell assay. **(I)** mRNA expression levels of *MMP2*, *MMP14*, and *MMP7* in SW620 cells, wild-type SW480 cells, and *SMARCA1*-knockdown SW480 cells, detected by quantitative PCR. Data were presented as mean ± standard error of the mean (*n* = 3 independent experiments). Statistical significance was determined by a two-tailed Student's *t*-test (for F, G, H, and I). ∗*p* < 0.05, ∗∗*p* < 0.01, ∗∗∗*p* < 0.001, and ∗∗∗∗*p* < 0.0001; ns, not significant.

Principal component analysis revealed high intra-group consistency within primary (SW480) and metastatic (SW620) cell populations (Fig. S1B). Using a fold-change cutoff of 1.5 and an FDR-adjusted *P* threshold of 0.05, we identified 13 differentially expressed proteins (7 up-regulated and 6 down-regulated) between the two groups, as shown in the volcano plot (Fig. 1B). The up-regulated proteins included *HDAC8*, *MDC1*, *SMYD2*, *CHEK1*, *BAZ1A*, *ARID2*, and *KMT5A*, while the down-regulated proteins comprised *SMARCA2*, *KDM5B, SMARCA1*, *SETD7*, *BRCA1*, and *BARD1*. To capture proteins with more moderate but potentially coordinated shifts in signaling regulation, we relaxed the criteria to a fold-change cutoff of 1.3 with a nominal *p* < 0.05, yielding 44 candidate proteins. Subsequent protein–protein interaction network analysis showed that these proteins were organized into three major functional clusters (Fig. S1C): chromatin remodeling factors (involving SWI/SNF and ISWI complexes), histone-modifying enzymes (methyltransferases/demethylases and acetyltransferases/deacetylases), and regulators of DNA damage response (including phosphorylation and ubiquitination pathways). These epigenetic modifiers are known to synergistically drive CRC progression through transcriptional reprogramming, tumor microenvironment remodeling, and genomic instability.^3^ Furthermore, functional enrichment analysis identified RNA polymerase II-mediated transcription as the most significantly enriched Gene Ontology (GO) biological process (Fig. S1D; Table S2), underscoring the central role of transcriptional dysregulation in the metastatic transition.

Proteomic analysis revealed significant down-regulation of *SMARCA1* (SNF2L), an ISWI chromatin remodeling complex member, in metastatic SW620 cells compared with primary SW480 cells (Fig. 1B; Fig. S2A). This differential expression pattern was further validated by Western blotting (Fig. S2B). *SMARCA1* has been predominantly studied in the context of neurogenesis, and its function in cancer biology remains largely unexplored. To evaluate the prognostic relevance of *SMARCA1* in CRC, we analyzed patient cohorts from the TCGA-COAD datasets. Kaplan–Meier survival analysis revealed that elevated *SMARCA1* expression was significantly associated with increased risk of both overall death (hazard ratio (HR) = 1.45, *p* = 0.00044, log-rank test) and recurrence (hazard ratio = 1.43, *p* = 0.0013, log-rank test) (Fig. 1C). Additionally, *SMARCA1* mRNA levels showed a positive correlation with advancing CRC pathological stages (Fig. 1D; Fig. S3A and S3B; Table S3). Levels were significantly elevated in stage III (Fig. S3A) and stage IV (Fig. 1D; Fig. S3B) compared with stage I. As stage III and IV denote regional and distant metastasis, respectively, these results directly associate heightened *SMARCA1* expression with metastatic progression and poorer clinical outcomes, supporting its role in enhancing CRC cell migratory potential.

These findings underscore the potential role of *SMARCA1* as a tumor driver and prognostic biomarker in CRC progression. Interestingly, its expression pattern suggests a stage-dependent regulatory function: while elevated *SMARCA1* levels in primary tumors are associated with poor prognosis, its expression is significantly reduced in paired metastatic cell lines. This bidirectional behavior implies that *SMARCA1* may act as a stage-specific modulator during CRC progression.

To investigate the functional role of *SMARCA1* in CRC metastasis, we performed CRISPR/Cas9-mediated knockdown of *SMARCA1* in SW480 primary tumor cells (Fig. 1E; Fig. S2C). *SMARCA1* depletion significantly inhibited cell proliferation in SW480 cells at 96 h (*p* < 0.05) (Fig. 1F). Moreover, SMARCA1 knockdown resulted in a marked reduction in cell migration and invasion, consistent with the low migratory and invasive capacities observed in SW620 cells (Fig. 1G, H). These findings reinforce the point that *SMARCA1* affects the development of CRC in a stage-dependent manner.

The tumor microenvironment, particularly its extracellular matrix, undergoes extensive remodeling during cancer progression, a process driven largely by matrix metalloproteinases (MMPs) that degrade extracellular matrix components to facilitate invasion and metastasis. Given the profound suppression of *SMARCA1* knockdown on migration and invasion in SW480 cells, we investigated its regulatory influence on MMP expression. *MMP1*, *MMP2*, *MMP7*, and *MMP14* are well-characterized mediators of tumor cell migration and invasion. Therefore, their expression levels were assessed following *SMARCA1* depletion. CRISPR/Cas9-mediated *SMARCA1* ablation in SW480 cells led to a significant down-regulation of *MMP2* and *MMP14*, accompanied by an up-regulation of *MMP7*, while *MMP1* remained unchanged (Fig. 1I; Fig. S4). Notably, this expression pattern closely mirrors that observed in the metastatic SW620 cells, which naturally exhibit low *SMARCA1* levels (Fig. 1I; Fig. S4), further supporting a regulatory link between *SMARCA1* and MMP-mediated metastatic behavior in CRC.

Previous studies in various cancers have established a correlation between the proteolytic activity of *MMP14* and activated *MMP2* with endothelial cell invasion and pericellular extracellular matrix degradation. These processes are associated with angiogenesis and metastasis, underscoring their potential as therapeutic targets.^4^ Conversely, *MMP7* expression increases in later stages of colon cancer and correlates with poor prognosis.^5^ Our findings suggest that during CRC progression, *SMARCA1* differentially regulates the expression of specific MMPs, potentially contributing to distinct aspects of tumor development at various stages. This stage-specific regulatory role highlights *SMARCA1*'s involvement in modulating the tumor microenvironment and underscores its potential as a context-dependent therapeutic target in CRC.

In conclusion, our study employed integrated data-dependent acquisition- and data-independent acquisition-mass spectrometry to profile epigenetic regulatory proteins in SW480 (primary) and SW620 (metastatic) CRC cells, identifying 13 differentially expressed proteins. Among these, the chromatin remodeling factor *SMARCA1* was significantly down-regulated in metastatic cells and found to regulate CRC cell migration and invasion by modulating matrix metalloproteinases (*MMP2*, *MMP7*, *MMP14*). Clinically, high *SMARCA1* expression in primary tumors was associated with poor patient prognosis, supporting its functional role in promoting tumor aggressiveness. This paradox, where *SMARCA1* appears oncogenic in early-stage tumors but is suppressed in metastatic cells, suggests a stage-dependent role for *SMARCA1* as an epigenetic modulator during CRC progression. This finding not only provides an epigenetic perspective to explain the phenotypic divergence between SW480 and SW620 cells but also lays a theoretical foundation for CRC metastasis intervention strategies targeting the *SMARCA1*–*MMP* axis.

## CRediT authorship contribution statement

**Chunqing Fu:** Writing – review & editing, Writing – original draft, Methodology, Investigation, Data curation, Conceptualization. **Shoufeng Duan:** Investigation, Formal analysis. **Chengwen Zhang:** Software, Investigation. **Xinyu Cui:** Investigation. **Fengxian Wang:** Investigation, Data curation. **Lichen Wang:** Investigation. **Jinglei Hu:** Investigation. **Tengjiao Li:** Investigation. **Lin Li:** Writing – review & editing, Writing – original draft, Supervision, Funding acquisition, Conceptualization.

## Funding

This study was supported by the 10.13039/501100001809National Natural Science Foundation of China (No. 22104084) and Startup funding from 10.13039/501100004921Shanghai Jiao Tong University.

## Conflict of interests

The authors declared no conflict of interests.
