# Supplementary material for: Proteomic profiling identifies SMARCA1 as a stage-specific epigenetic regulator of colorectal cancer metastasis through MMP modulation
Source: Genes Dis. 2025 Dec 24;13(6):102006. doi: 10.1016/j.gendis.2025.102006 (PMC13355020; doi:10.1016/j.gendis.2025.102006)
Supplement: Multimedia component 1 [file mmc1.docx]

# Methods

## Cell Culture

SW480, SW620 and HEK293T cell lines were maintained in Dulbecco’s Modified Eagle Medium (DMEM, Thermo Fisher Scientific) supplemented with 10% fetal bovine serum (FBS, Adamas life, China) and penicillin/streptomycin (PS, 100 unit/mL) in a humidified atmosphere with 5% CO2 at 37 °C, and the culture medium was changed every 2 to 3 days as necessary.

## Plasmid construction

In order to establish a mass spectrometric library of low-abundance proteins, the full-length complementary human cDNAs of *ATAD2*, *BRD7*, *BRD1*, *BRPF1*, *PBRM1*, *DPF3*, *KAT6B*, *MLLT3*, *ING4*, *ATR*, *UBE2B*, *RNF2*, *BAP1*, *L3MBTL1*, *POLL*, *KDM3A*, *KDM4C*, *KDM4D*, *KDM5A*, *KDM5C*, *PSMA1*, *DOT1L*, *KMT5A*, *SETMAR*, *SMYD2*, *SETD7*, *PRDM3*, *CCNY*, *PAXIP1*, *SGF29*, *EHMT2*, *BDNF*, *CBX7*, *NBN*, *ORC1*, *JMJD6*, *PARP1*, *NEDD8*, *KAT8*, *HDAC3*, *SIRT1*, *USP3*, *USP15*, *USP22*, *BRCA1*, *BARD1*, *JAK2*, *RNF20*, *RNF40*, *ZMYND11*, *KDM3B*, *KDM5D*, *SETDB1*, *KDM6A*, *CARM1*, *MORF4L1*, *SIRT6*, *SIRT3*, *SIRT7*, *SIRT2*, *HDAC4*, *PHF8*, and *METTL9* were amplified using PCR technique and then cloned into the pFL3-flag vector, separately.

CRISPR-Cas9 system was carried out to decrease protein expression of *SMARCA1*. Three sgRNA sequences targeting *SMARCA1* designed by CHOPCHOP (<http://chopchop.cbu.uib.no/>) and SYNTHEGO (<https://design.synthego.com>) website were 5’-GATAGTGGCGGTCGCATCCG-3’ (PAM CGG), 5’-GGATGCGACCGCCACTATCG-3’ (PAM TGG), and 5’-TTCAAATCTCTTTGCTCGGT-3’ (PAM CGG). These sequences were cloned into the pLV3-U6-MCS-sgRNA-Cas9-EGFP-Puro vector. Three plasmids were co-transfected into SW480 cell using Lipo8000 (Beyotime, China) in accordance with the protocol. Transfected cells were screened using 2 μg/mL puromycin to obtain the SW480 cell line with stable knockdown of the *SMARCA1*.

## Expression and purification of low abundance chromatin regulatory proteins

To obtain a mass library of low abundance proteins, each overexpression pFL3-flag plasmid containing CDS sequence was transfected into HEK293T cells using Lipo293 transfection reagent (Beyotime, China). All cells were collected after 48 h, and proteins were extracted using RIPA lysis buffer (50 mM Tris (pH 7.4), 150 mM NaCl, 1% TrionX-100 (v/v), 1% sodium deoxycholate (w/v), 0.1%SDS (w/v), and protease inhibitor cocktail). Flag-tagged proteins were immunoprecipitated using anti-flag magnetic beads (SB-PR002, ShareBio, China) according to manufacturer’s instructions to obtain pure low-abundance proteins.

## Chromatin Isolation assay

To assess the differential expression of chromatin regulatory proteins in SW480 and SW620 cells, chromatin fraction was obtained according to the method described by Juri Rappsilber’s group. ^[1](#_ENREF_1)^ In brief, approximately 1 × 10^7^ cells were crosslinked using 1% formaldehyde, followed by termination of crosslinking using 0.25 M glycine. After PBS washing, cells were collected and lysed using cell lysis buffer (25 mM Tris (pH 7.4), 0.1% Triton X-100 (v/v), 85 mM KCl and protease inhibitor cocktail). Rnase A (Sangon, China) at 200 μg/ml was added to the solution to remove RNA. Cell nuclei were lysed using SDS buffer (50 mM Tris (pH 7.4), 10 mM EDTA, 4% SDS (w/v) and protease inhibitor cocktail) and solubility was increased using a 3-fold volume of urea buffer (10 mM Tris (pH 7.4), 1 mM EDTA and 8 M urea) to remove proteins not covalently bound to DNA. Subsequently, urea was washed using SDS buffer. Finally, the chromatin precipitate was resuspended using storage buffer (10 mM Tris (pH 7.4), 1 mM EDTA, 25 mM NaCl, 10% glycerol (v/v) and protease inhibitor cocktail), sonicated for 15 min at 10s-on,10s-off, 5% power (JY92-IIDN, ScientZ, China) to obtain chromatin proteins, and quantified using a BCA kit (ShareBio, China).

## Peptide Sample Preparation for Mass Spectrometry (MS)

For each sample, 100 μg of protein was mixed with SDS loading buffer (50 mM Tris‐HCl (pH 6.8), 2% SDS (w/v), 10% Glycerol (v/v), 1% β-Mercaptoethanol (v/v), and 0.1% bromophenol blue (w/v)) and incubated at 95 °C for 30 min to reverse crosslink. All samples were run in 10% SDS‐PAGE gels until the dye front was 1 cm from the bottom for higher reproducibility. The gels were washed with deionized water and decolorized using decolorizing solution (25 mM NH_4_HCO_3_ and 50% acetonitrile (ACN)). Each lane was cut into cubes of ≈ 1 mm^2^ and dehydrated with ACN for additional 10 min with shaking. Dehydrated gel pieces were reduced with 10 mM dithiothreitol (DTT, Adamas, China) at 37°C for 60 min and then dehydrated with ACN. Alkylation was conducted by replacing the DTT solution with 25 mM iodoacetamide (IAA, Sigma-Aldrich) and incubated at room temperature for 20 min in the dark followed by dehydrating with ACN. The gel pieces were digested with 2 ng/μL of trypsin (Thermo Fisher Scientific) in 25 mM NH_4_HCO_3_ overnight at 37 °C. After digestion, the supernatant was collected to obtain the peptides. To increase the yield of peptides, the supernatant containing peptides were further extracted by adding using 65% ACN and 5% formic acid (FA) to the gel and sonicated for 5 min in a water bath and incubated at 37°C for 30 min. ACN was added for the same extraction process as before and the supernatant was collected. All the supernatants were combined and dried in a vacuum centrifugal dryer (Thermo Fisher Scientific) at 50°C and resuspended in 100 μL of 0.1% formic acid. All samples were desalinated using Empore StageTips (6091, CDS Analytical, US).

## LC-MS/MS Analyses

The dried peptide samples were re-dissolved with 0.1% FA and further analyzed by electrospray liquid chromatography-tandem mass spectrometry using an Orbitrap Exploris 480 (Thermo Fisher Scientific) coupled to an EASY-nLC 1200 (Thermo Fisher Scientific). Samples were run on a C_18_ Column (3 μm particle size, 75 μm × 20 cm, 100 Å) at a flow rate of 250 nL/min using the following gradient: 0 to 5 min 2 to 8% B, 5 to 97 min 8 to 22% B, 97 to 110 min 22 to 35% B, 110 to 111 min 35 to 90% B, 111 to 120 min at 80% B. Mobile phase A was 0.1% FA in water and Mobile phase B was 0.1% FA in 80%ACN. DDA MS parameters were set to: (1) MS: scan range (m/z), 350-1,500; orbitrap resolution, 60,000; time (ms), 20; normalized AGC target (%), 300; number of dependent scans, 30; exclusion duration, 30 s; exclude after n times, 1. (2) HCD-MS/MS: orbitrap resolution, 15,000; time (ms), 20; normalized AGC target (%), 100; HCD CE (%), 30; include charge exclusion, 2-6; 1; 1.0 m/z isolation window. The same nano-LC system and gradient are used for DIA analysis. DIA MS parameters were set to: (1) MS: scan range (m/z), 350-1,500; orbitrap resolution, 60,000; ACG target, standard; cycle time (s), 3. (2) HCD-MS/MS: orbitrap resolution, 30,000; scan range (m/z), 200-2000, time (ms), 54; normalized AGC target (%), 1000; HCD CE (%), 32; 60 DIA isolation windows of 10 *m/z* from m/z 400-1000. The DDA data were searched using MaxQuant (version 2.4.9), and then the search data were imported into Skyline software (version 23.1) to create a mass spectral library of 271 chromatin regulatory proteins. The DIA data were imported into Skyline, and the chromatograms of the peptides of the related proteins were extracted for quantification and comparison.

To assess the overall variance of the data obtained from skyline analysis, principal component analysis was performed using fast.prcomp function in R. Correlation clustering of samples was performed using Pearson correlation coefficient. Differentially expressed proteins (DEPs) were identified using the limma package in R (version 4.3.0). DEPs were defined as proteins with |fold change| ≥ 1.5 and Benjamini-Hochberg FDR‑adjusted *p* < 0.05; results are visualized in a volcano plot. For exploratory functional analyses, a relaxed cutoff of |fold change| ≥ 1.3 with nominal *p* < 0.05 was also used to capture additional candidate proteins for downstream gene‑set and pathway enrichment. Cluster analysis of DEP was plotted using the pheatmap function in R and the clustering method was the Euclidean distance method. GSEA enrichment (GO) and signaling pathway (KEGG) analysis of DEP were performed using the clusterProfiler package in R. Protein interaction network analysis (PPI) of DEP was carried out using the STRING database with a confidence level set to 0.7 and the result was furthered visualized by Cytoscape (Version 3.10.3).

## Quantitative real-time PCR (RT-qPCR)

Eastep Super Total RNA Extraction Kit (LS1040, Promega) was employed to extract total RNA following manufacturer’s instructions. The mRNA levels were assessed using HiScript III RT SuperMix for qPCR (+gDNA wiper) (R323, Vazyme, China) and 2×Universal SYBR Green qPCR Premix (Q312-02, Vazyme, China). All results were normalized to *GAPDH* expression. The relative expression of mRNAs was quantified using the 2^–∆∆Ct^ method. Primer sequences used were: *GAPDH*, forward (F) 5’-GTCGGAGTCAACGGATTTGG-3’, reverse (R) 5’ - TGCCATGGGTGGAATCATATTG-3’; *MMP1*, F 5’-AGAGCAGATGTGGACCATGC-3’, R 5’- TTGTCCCGATGATCTCCCCT-3’; *MMP2*, F 5’-GTCCCCATGAAGCCCTGTTC-3’, R 5’- CCCTGGAAGCGGAATGGAAA-3’; *MMP7*, F 5’- AACAATTGTCTCTGGACGGC-3’, R 5’- TCTCTTGAGATAGTCCTGAGCCT-3’; *MMP14*, F 5’-GCGTCCATCAACACTGCCTA-3’, R 5’- CACCCAATGCTTGTCTCCTTTG -3’.

## Protein extraction and western blotting

Cells were directly harvested with SDS loading buffer (50 mM Tris‐HCl (pH 6.8), 2% SDS (w/v), 10% Glycerol (v/v), 1% β-Mercaptoethanol (v/v), and 0.1% bromophenol blue (w/v)). Protein expression was detected by Western blotting. Equal amounts of proteins were fractionated by 12% sodium dodecyl sulfate (SDS)-polyacrylamide gel electrophoresis. Anti-*SMARCA1* (A10248, ABclonal, China) and Anti-GAPDH (SB-AB0037, ShareBio, China) were used and the expression signal was amplified by incubating the secondary antibody of the corresponding species. The blots were visualized using ECL chemiluminescence solution (SB-WB012, ShareBio, China) on a ChemiDoc Imaging Systems (BioRad). Grayscale values of the target bands were calculated using ImageJ for quantitative comparison.

## Wound healing assay

Differences in migration rates among SW620, wild type and *SMARCA1*-knockdown SW480 cells were assessed using a wound healing assay. Cells were seeded and gently scraped with a 10 μL sterile pipette tip when the cells achieved 90% confluence. The wounded cells were continuously cultured for 48 hours. Wound closure was recorded at 0 and 48 h under the IX73 inverted fluorescence microscope (Olympus Corporation, Japan). The migration rate was measured by the ratio of the cell area of day 0 and day 2 measured by ImageJ.

## Transwell assays

To assess cell invasion, the transwell membrane (6.5 mm in diameter with 8.0 µm pores, Corning, USA) were coated with a 300 ng/µL Matrigel solution (082704, ABW, Shanghai Nova Pharmaceutical Technology, China) according to manufacturer’s instructions. 1 × 10^5^ SW620, wild type and *SMARCA1*-knockdown SW480 cells were seeded into the inner chamber in serum-free medium, and the outer chamber was filled with medium containing 10% FBS. After 72 h of incubation, non-invaded cells at the inner bottom of the chamber were scraped off using a cotton swab, and invaded cells at the outer bottom of the chamber were fixed with methanol for 15 min and stained with 0.1% crystal violet for another 15 min. Then 5 fields were selected and photographed using an BX53 upright fluorescence microscope (Olympus Corporation, Japan). The invasion differences of different cells at 72 h were evaluated by measuring the stained cell area using ImageJ.

## Online Data Analysis

The prognostic effect of *SMARCA1* expression in CRC patients were analyzed using the Kaplan-Meier Plotter online website, including overall survival analysis (OS) and Recurrence free survival (RFS). ^[2](#_ENREF_2)^

SMARCA1 mRNA expression data across different clinical stages for the TCGA, CPTAC‑2, and Sidra‑LUMC AC‑ICAM cohorts were downloaded from the cBioPortal platform. ^[3-5](#_ENREF_3)^ For TCGA, colon adenocarcinoma (COAD) and rectum adenocarcinoma (READ) were combined into a single TCGA colorectal cancer cohort (COAD and READ). Clinical sub‑stages (e.g., I, IA, IB; II, IIA, IIB; etc.) were collapsed into their parent stage (all IA/IB to Stage I; IIA/IIB to Stage II; etc.) prior to groupwise analysis; merged per‑stage sample counts are reported in the Results and figure legends. Expression values were log2‑transformed before statistical testing and visualization. Normality within each stage group was evaluated using the Shapiro–Wilk test, and homogeneity of variances across stages was evaluated using Levene’s test. The choice between parametric and nonparametric tests was determined by these diagnostics. For CPTAC‑2, where diagnostic tests indicated that parametric assumptions were met, group differences were assessed by one‑way ANOVA with Tukey’s honestly significant difference (HSD) post hoc test; Tukey‑adjusted *p* values are reported. For the Sidra‑LUMC AC‑ICAM cohort and the combined TCGA cohort, overall differences across stages were assessed by the Kruskal–Wallis H test. When the Kruskal-Wallis test was significant, pairwise comparisons were performed using Dunn’s test. All pairwise *p* values from Dunn’s tests were adjusted for multiple comparisons using the Benjamini–Hochberg false discovery rate (FDR) procedure and reported as adjusted *p* values (FDR-adjusted *p*). Statistical significance was defined as two‑sided FDR-adjusted *p* < 0.05.

# References

1. Kustatscher G; Wills KL; Furlan C, et al. Chromatin enrichment for proteomics. *Nat. Protoc.* 2014; 9 (9): 2090-9.

2. Gyorffy B. Integrated analysis of public datasets for the discovery and validation of survival-associated genes in solid tumors. *Innovation. (Camb).* 2024; 5 (3): 100625.

3. Cerami E; Gao J; Dogrusoz U, et al. The cBio cancer genomics portal: an open platform for exploring multidimensional cancer genomics data. *Cancer Discov*. 2012; 2 (5): 401-4.

4. de Bruijn I; Kundra R; Mastrogiacomo B, et al. Analysis and Visualization of Longitudinal Genomic and Clinical Data from the AACR Project GENIE Biopharma Collaborative in cBioPortal. *Cancer Res*. 2023; 83 (23): 3861-3867.

5. Gao J; Aksoy BA; Dogrusoz U, et al. Integrative analysis of complex cancer genomics and clinical profiles using the cBioPortal. *Sci Signal*. 2013; 6 (269): pl1.

# Supplementary Figures


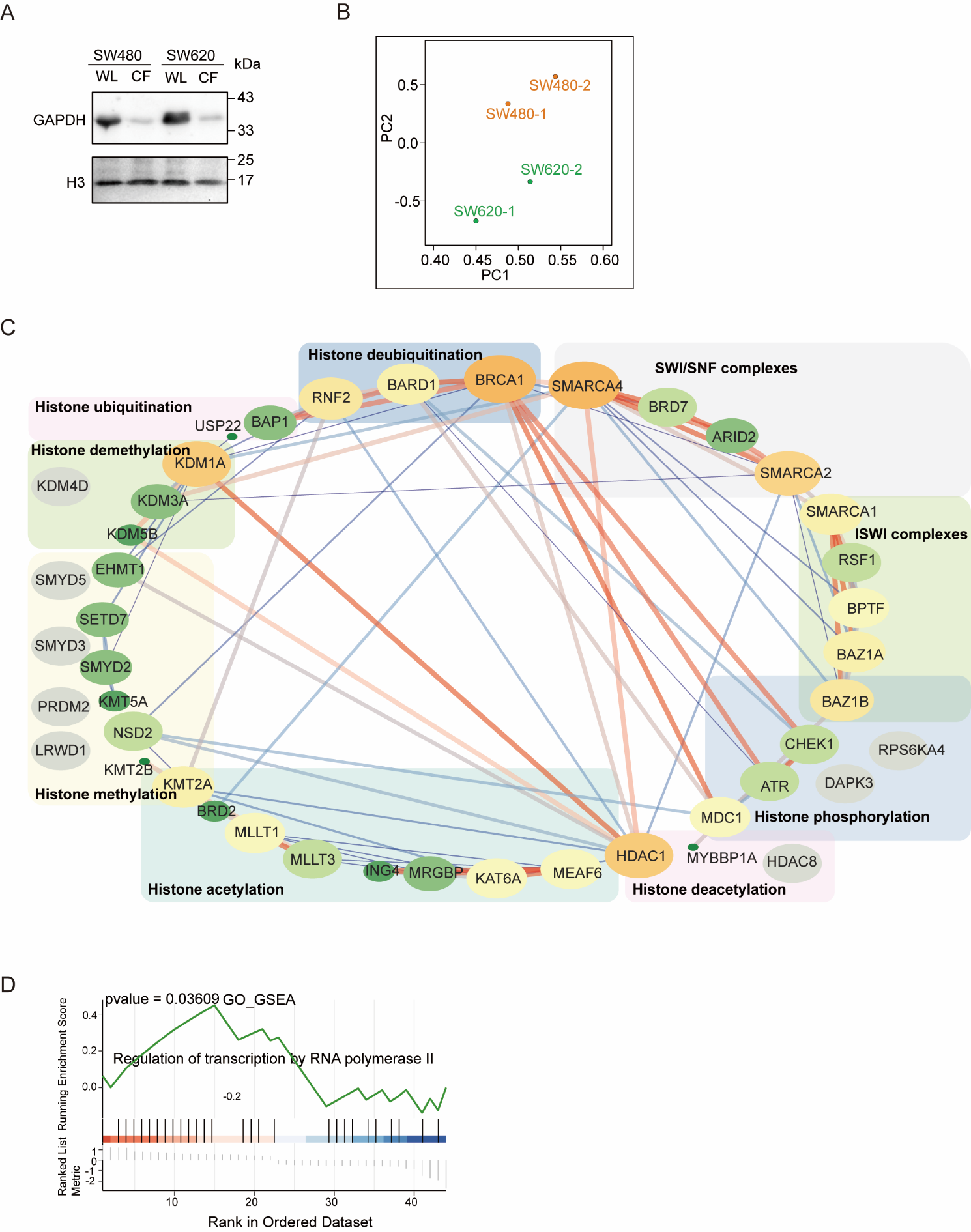


**Figure S1.** Comparative proteomic profiling of chromatin-associated proteins between metastatic (SW620) and primary (SW480) colorectal cancer cell lines. **(A**) Chromatin separation effects of SW480 and SW620 shown by western blotting. WL, whole lysate. CF, chromatin fraction. **(B)** Principal component analysis (PCA) of chromatin-associated proteins in paired CRC cell lines SW480 (primary tumor) and SW620 (lymph node metastasis) using data-independent acquisition (DIA) mass spectrometry. **(C)** Protein-protein interaction network of metastasis-associated regulators (fold change >1.3, *p* < 0.05) reconstructed using STRING database (minimum confidence score: 0.7). Node size reflects interaction degree centrality, while edge thickness corresponds to association strength. **(D)** GO biological processes identified through functional enrichment analysis (GSEA) with *p* < 0.05.


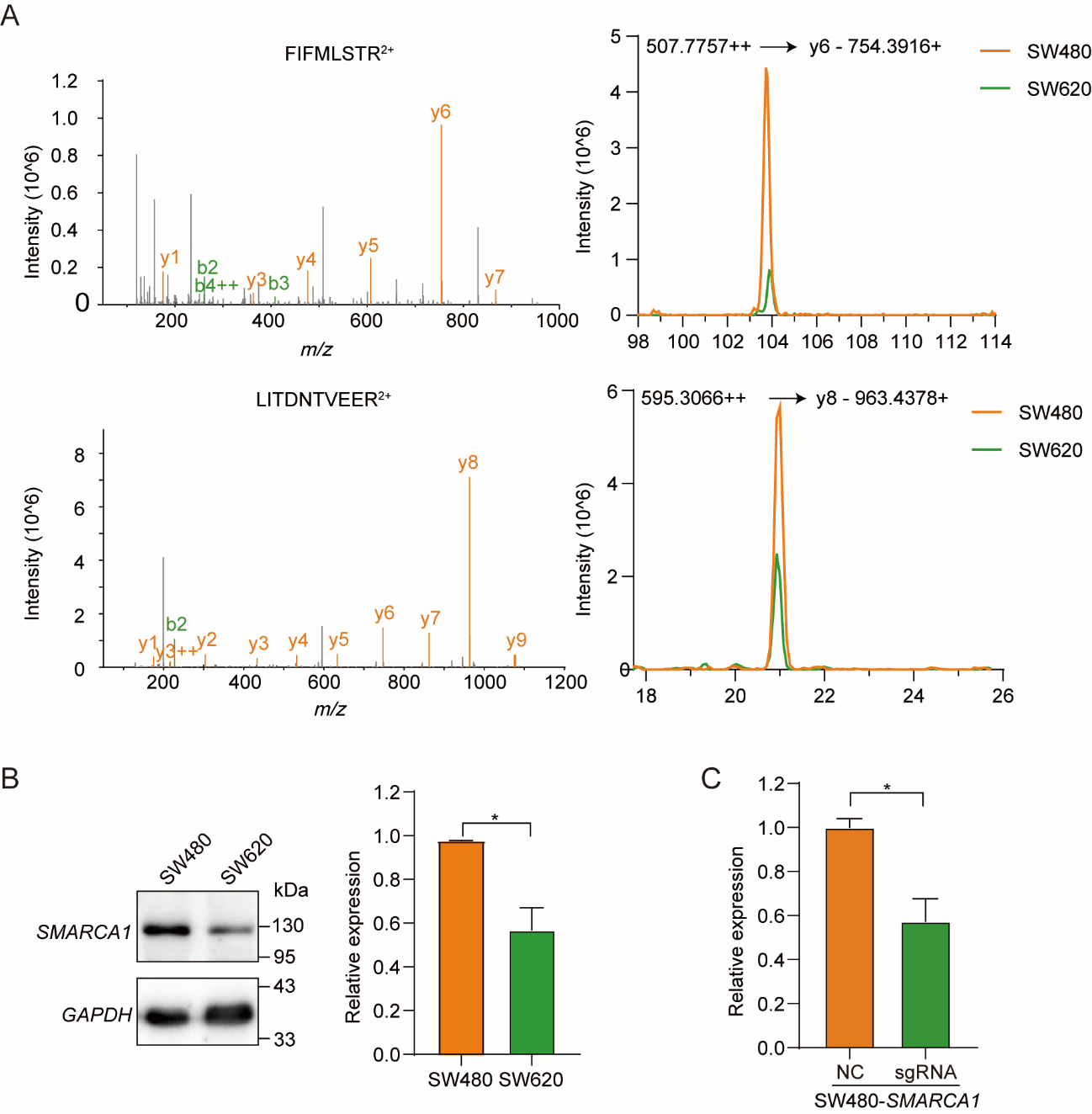


**Figure S2.** Identification of *SMARCA1* as a differentially expressed protein between SW480 and SW620 cell lines, and was successfully knocked down in SW480 cells. **(A)** Representative MS/MS spectra (left panel) and extracted ion chromatograms (right panel) of two peptides (m/z indicated) unique to *SMARCA1* in SW480 (orange) versus SW620 (green) chromatin fraction. **(B)** Western blot validation of *SMARCA1* expression differences between paired cell lines. Quantification normalized to *GAPDH* loading control. Data represent mean ± SEM. * *p* < 0.05. **(C)** *SMARCA1* knockdown in SW480 cells was achieved using the CRISPR/Cas9-sgRNA system and verified by Western blot. Data are presented as mean ± SEM (n = 3 independent experiments). Statistical significance was determined by two-tailed Student’s t-test. **p* < 0.05.


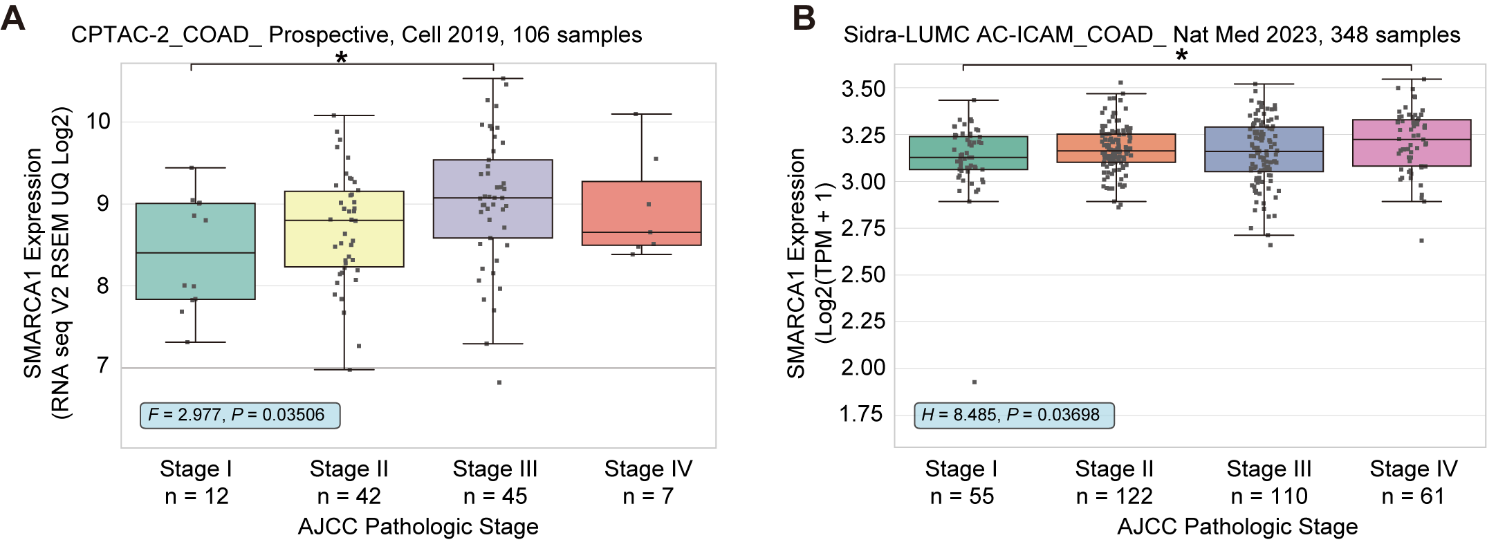


**Figure S3.** Expression analysis of *SMARCA1* in colorectal cancer patients and cell models from public datasets. **(A)** *SMARCA1* mRNA expression across different stages of colorectal cancer from the CPTAC-2 cohort. Statistical significance was assessed by one-way ANOVA with Tukey’s multiple‑comparisons post hoc test. **(B)** *SMARCA1* mRNA expression across colorectal cancer stages from the Sidra-LUMC AC-ICAM cohort. Differences were evaluated by the Kruskal-Wallis test with Dunn’s post hoc pairwise comparisons. Pairwise *p* values were adjusted using the FDR method. * FDR-adjusted *p* < 0.05.


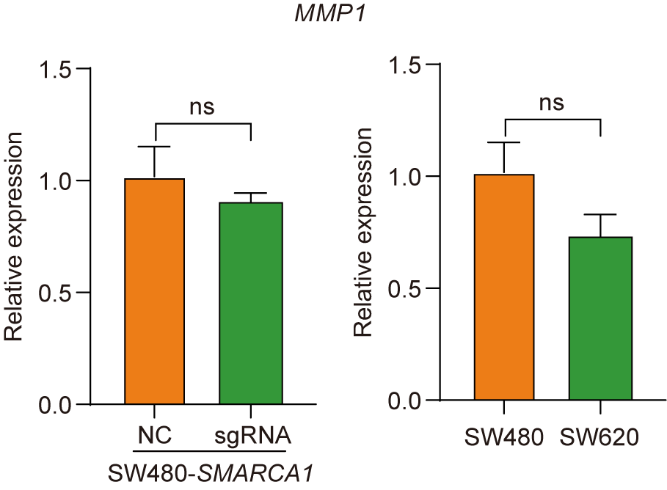


**Figure S4.** mRNA expression levels of *MMP* in SW620, wild-type SW480, and *SMARCA1*-knockdown SW480 cells, detected by qPCR assay. Data are presented as mean ± SEM (n = 3 independent experiments). Statistical significance was determined by two-tailed Student’s t-test. Ns, not significant.
